# Postinfarction Ventricular Septal Ruptures During the COVID-19 Pandemic: Two Case Series

**DOI:** 10.7759/cureus.40331

**Published:** 2023-06-12

**Authors:** Nkechi A Okam, Jonathan Vargas, Mohamed Zakee Mohamed Jiffry, Mohammad A Ahmed-Khan, Felipe Carmona Pires, Uzochukwu Ibe

**Affiliations:** 1 Department of Internal Medicine, Danbury Hospital, Yale School of Medicine, Danbury, USA; 2 Department of Internal Medicine, University of Vermont, Burlington, USA; 3 Department of Cardiology, Danbury Hospital, Yale School of Medicine, Danbury, USA

**Keywords:** post-myocardial infarction ventricular septal rupture, ventricular septal rupture, myocardial infarction, ventricular septal defect (vsd), covid 19, post mi vsd

## Abstract

This case series highlights the occurrence of hemodynamically significant ventricular septal defects (VSDs) in two patients presenting with ST-elevation myocardial infarction (STEMI) during the COVID-19 pandemic. This paper aims to emphasize the delayed presentation of cardiac emergencies, such as STEMI, due to concerns about contracting COVID-19. This delay has led to an increased risk of rare complications, including VSD, associated with STEMI. The first case involves a 92-year-old male with a history of hypertension, hyperlipidemia, chronic kidney disease, and coronary artery disease. He presented with acute chest pain, and diagnostic tests revealed ST elevations and a VSD. Despite intervention efforts, including hemodynamic support, the patient's condition deteriorated, and he passed away due to advanced age and high surgical risk. The second case involves a 62-year-old female with a medical history of diabetes, hypertension, and hyperlipidemia. She presented with left-sided chest pain, and an angiogram revealed a mid-right coronary artery stenosis and a thrombus. During the procedure, the patient experienced hypotension, requiring hemodynamic support. Subsequent evaluations identified a large VSD with right ventricular dysfunction. The patient underwent a series of interventions, including a ventricular assist device and VSD closure, but experienced multi-organ failure and ultimately passed away. VSDs following acute myocardial infarction (MI) are rare but life-threatening complications. Early revascularization is crucial in preventing the development of VSDs. These cases demonstrate the importance of prompt diagnosis and intervention, as delayed presentation increases the risk of mechanical complications. Surgical closure remains the definitive treatment for postinfarction VSDs.

## Introduction

COVID-19, caused by SARS-CoV-2, is responsible for the current pandemic, killing more than 936,001 people as of September 16, 2020 [1]. COVID-19 has been found to have worse outcomes in individuals with comorbid chronic heart and lung disease. As new data continues to emerge, the cardiovascular complications related to COVID-19 are more evident. The rise in COVID-19 hospitalizations was also associated with a 30% to 40% decline in visits for cardiovascular emergencies, including ST-elevation myocardial infarction (STEMI), stroke, and heart failure [2], and as a result, patients often presented to the hospital later than expected.

## Case presentation

Case 1

**Figure 1:**
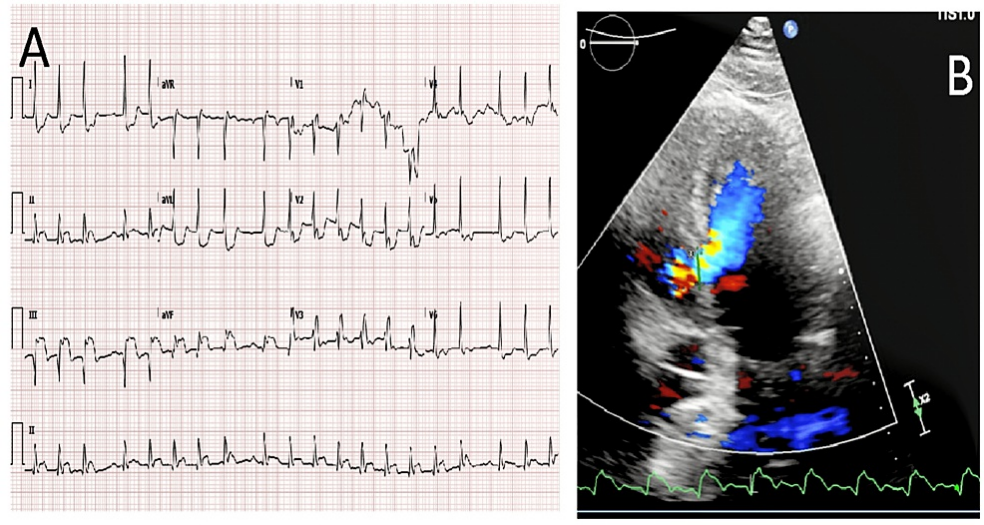
(A) EKG showing a sinus rhythm with Morbitz II second-degree AV block and inferior ST elevation with contralateral ST depression in the lateral leads; (B) EKG showing a ventricular septal defect at the inferobasal segment measuring 1.2 cm in length. AV, atrioventricular; EKG, echocardiogram

A 92-year-old male presented with acute chest pain that started three days before arrival. He had a medical history significant for hypertension, hyperlipidemia, chronic kidney disease, and coronary artery disease with three-vessel bypass grafting. A clinical exam revealed normal heart sounds without murmurs and clear lungs. Their blood pressure was 126/60 mmHg. Echocardiogram (EKG) showed sinus tachycardia with second-degree atrioventricular (AV) block and ST elevations in the inferior leads. He was treated with aspirin and ticagrelor and taken to the cardiac catheterization lab. An emergent angiogram showed predominantly left main and right coronary artery (RCA) disease. The vein grafts to his obtuse marginal and RCA were occluded. A right heart catheterization showed a significant left to right shunt, and a ventriculogram showed a ventricular septal defect. The right atrial saturation was 60.7%, and the pulmonary artery saturation was 91%. An Impella and an intra-aortic balloon pump (IABP) were inserted for hemodynamic support, left ventricular (LV) unloading, and to augment coronary perfusion. A transthoracic EKG showed a 1.2 cm ventricular septal defect in the inferobasal segment with a left to right shunting. The pulmonary-to-systemic-shunt ratio (Qp:Qs) was 1.9, and the LV ejection fraction was 70% to 75%. On hospital day 2, the patient became hypotensive with blood pressures in the 78/45 to 96/48 mmHg range with worsening signs of perfusion, including rising lactic acid level to 3 mmol/L and creatinine levels 2.9 mg/dL (baseline of 1.7 mg/dL). He was evaluated by the cardiothoracic surgical team and felt to be a poor surgical candidate owing to advanced age, hemodynamic instability, and high surgical risk. He continued to deteriorate and ultimately passed away after his family decided to stop aggressive measures (Figure 1).

Case 2

**Figure 2:**
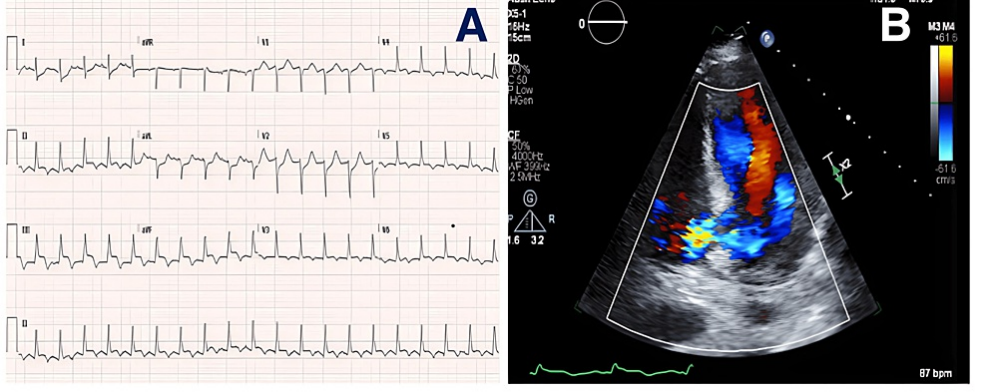
(A) EKG showing inferior ST elevation; (B) EKG showing an inferobasal ventricular septal defect with left to right shunting. EKG, echocardiogram

A 62-year-old female presented with left-sided chest pain that started six hours before arrival. She had a medical history significant for non-insulin-dependent diabetes, hypertension, and hyperlipidemia. A clinical exam revealed normal heart sounds and a 2+ systolic murmur heard best near the apex. Her blood pressure was 101/66 mmHg and her heart rate was 107 beats per minute. EKG showed ST elevations in the inferior leads. She was treated with aspirin and ticagrelor and taken to the cardiac catheterization lab. An angiogram showed a mid-RCA stenosis for which a drug-eluting stent was implanted. There was a distal RCA lesion that had a filling defect consistent with a thrombus that was treated with balloon angioplasty. During the procedure, the patient was hypotensive and required IV norepinephrine. An arterial line was used to monitor blood pressure. Post-procedure, she remained hypotensive and Neo-Synephrine was added to maintain hemodynamic stability. An EKG showed an ejection fraction of 70% to 75% with inferior wall hypokinesis and dilated and hypokinetic right ventricle. A repeat exam was remarkable for a loud holosystolic murmur at the left lower sternal border. Swan-Ganz catheter was then inserted for further hemodynamic assessment. Pulmonary capillary wedge pressure was 28 mmHg with a central venous pressure of 15 mmHg. There was a step up in venous saturations from 49% proximally to 79% distally, suggesting a shunt at the ventricular level. A repeat EKG showed a large VSD in the basal aspect of the inferoseptum with a severe decrease in right ventricular function. Laboratory investigation showed worsening kidney function, hyperkalemia, and acidosis. She was subsequently intubated for airway protection and taken to the operating room for a biventricular assist device (BIVAD). The post-procedure course was complicated by multiorgan failure requiring continuous veno-venous hemofiltration (CVVH), disseminated intravascular coagulation, and anemia requiring transfusion of blood products. BIVAD flows were poor as the patient developed tamponade physiology for which a pericardial window was performed. She was maintained on the BIVAD, pressor, inotropic support, and CVVH and remained stable. She developed heparin-induced thrombocytopenia for which her aspirin and ticagrelor were stopped and bivalirudin was initiated. A transesophageal EKG was done to assess VSD ahead of Amplatzer versus surgical closure. It measured 15 m × 13 m with a hyperdynamic left ventricle and a mildly depressed RV function. She underwent a VSD closure using the Amplatzer closure device on hospital day 15. A right ventricular assist device was inserted. Postoperatively, her clinical course deteriorated over the next few days with rising lactic acid levels and a gastrointestinal bleeding episode. Later that day, she became asystolic and subsequently passed away (Figure 2).

## Discussion

Ventricular septal ruptures (VSRs) are an uncommon and life-threatening complication following an acute myocardial infarction (MI). There has been a decrease in the incidence of VSR during the pre-reperfusion era from 1% to 2% to 0.17% to 0.31% after primary percutaneous interventions [3,4]. Timing of revascularization is important in preventing the development of VSR by salvaging the myocardium and limiting infarct expansion [3]. The risk of mechanical complications is increased with late reperfusion [5]. Pathophysiologically, VSR is associated with a transmural infarction resulting from a complete occlusion of any coronary artery that supplies the septum [6]. Typically, VSR occurs within three to five days on the index MI. Risk factors for developing postinfarction VSRs include older age, female gender, prior stroke, ST-segment elevation, elevated cardiac markers, hypotension, and delayed or no reperfusion [5,6].

Anatomically, coronary artery involvement usually correlates to the location and complexity of the VSR. Anterior infarctions usually result in apical VSRs, whereas inferior infarctions result in more complex ruptures in the basal inferoposterior septum [4] as was the case in our patients. VSR results in left-to-right shunting, leading to right ventricular volume and pressure overload. The degree of shunting is determined by the shunt fraction (Qp/Qs) [3]. These patients are at an increased risk of cardiogenic shock, depending on the size of the infarction, degree of shunting, and RV dysfunction.

Transthoracic and Doppler's echocardiography are crucial in establishing the diagnosis and assessing the hemodynamic impact of VSR. Surgical closure is the definitive treatment of postinfarction VSRs [3]. Data from the Society of Thoracic Surgeons National database found an overall in-hospital stay or 30-day mortality of 42.9% with a decrease in mortality with delayed repair [7]. Risk factors for operative mortality include age, female gender, shock, inferior infarction, preoperative IABP use, preop dialysis, mitral insufficiency, emergent surgery, and timing of repair [7]. The optimal timing of VSR closure is still controversial.

According to the American College of Cardiology guidelines, emergent surgical repair is recommended regardless of hemodynamic status. There is a tendency for delayed surgery in the setting of a VSR. This is due to evidence that metalloproteinase activity and tissue breakdown peak by day 7, whereas deposition of new collagen begins by day 2 to day 4 [8]. Necrotic myocytes are replaced by collagen by day 28, and this potential delay in repair allows the reorganization of friable tissue to facilitate repair [3,8]. VSRs are associated with a high mortality risk and a poor prognosis. Once a diagnosis has been established, aggressive measures are usually employed to maintain hemodynamic stability and ensure adequate tissue perfusion before closing the VSR. The use of mechanical support devices allows for delayed repair, but this is usually dependent on the patient’s clinical course.

## Conclusions

In conclusion, both patients with multiple cardiovascular comorbidities presented with STEMI complicated by hemodynamically significant VSR. They subsequently underwent left and right cardiac catheterization. Despite aggressive interventions in both cases, the patient's clinical course was complicated by multiple organ failures and ultimately resulted in poor outcomes. These cases shed light on a potential contributing factor to the late presentation of patients with multiple cardiovascular comorbidities and the subsequent development of less common complications, such as hemodynamically significant VSR. The standard of care measures in place normally prevent such complications from occurring. However, the unprecedented challenges posed by the ongoing pandemic may have impeded timely access to medical care for these individuals, leading to delays in seeking medical attention. As a result, the patients experienced adverse outcomes that were not typical under normal circumstances. This highlights the need for heightened awareness and timely management of cardiovascular emergencies in the context of the ongoing pandemic.
